# Registration-based 3D Light Sheet Fluorescence Microscopy and 2D histology image fusion tool for pathological specimen

**DOI:** 10.1038/s41598-026-57893-5

**Published:** 2026-06-30

**Authors:** Marcel Brettmacher, Philipp Nolte, Diana Pinkert-Leetsch, Felix Bremmer, Jeannine Missbach-Guentner, Christoph Rußmann

**Affiliations:** 1 University of Applied Sciences and Arts Hildesheim/Holzminden/Goettingen, Von-Ossietzky-Straße 99, Goettingen, 37085 Lower Saxony Germany; 2 Faculty of Mathematics and Computer Science, Georg-August University School of Science, Goldschmidtstr. 7, Goettingen, 37077 Lower Saxony Germany; 3 Institute for Clinical and Interventional Radiology, University Medical Center, Robert-Koch-Straße 40, Goettingen, 37075 Lower Saxony Germany; 4 Institute for Cardiac Imaging, University Medical Center, Robert-Koch-Straße 40, Lower Saxony 37075 Goettingen, Germany; 5 Institute of Pathology, University Medical Center, Robert-Koch-Straße 40, Goettingen, 37075 Lower Saxony Germany

**Keywords:** Image registration, Multimodal fusion, Histology, LSFM imaging, Pathology, Biological techniques, Computational biology and bioinformatics, Engineering, Mathematics and computing

## Abstract

Histological analysis traditionally relies on thin tissue sections, providing inherently two-dimensional (2D) information. However, this approach captures only a fraction of the entire sample and lacks the spatial context necessary for comprehensive tissue assessment. Recent advancements in multimodal imaging have introduced the fusion of histological data with three-dimensional (3D) imaging techniques, such as Light Sheet Fluorescence Microscopy (LSFM), to enhance tissue analysis by integrating complementary spatial information. A key challenge in this fusion process is the accurate alignment of corresponding structures across modalities, which is complicated by differences in resolution, sectioning-induced deformations, and varying imaging orientations. This is further complicating in the case of 2D-to-3D registration where the initial alignment of the image inside the volume is unknown and registration processes are computationally expensive due to six degrees of freedom in the placement. Here, existing solutions often require manual selection of image pairs, fiducial markers or technical expertise, limiting accessibility to non-specialist users. To address these limitations, we introduce LitSHi (Light Sheet meets Histology), a novel registration tool that enables the automated and precise alignment of LSFM and histological images. LitSHi allows multimodal image fusion to be performed fully automatically, which significantly reduces the need for manual intervention. Using testicular tumor and pancreatic specimens, we evaluated LitSHi and demonstrated its ability to enhance structural correspondence between LSFM and histological images. The automated registration process markedly improved both efficiency and alignment accuracy compared with conventional manual or semi-automated approaches. Overall, LitSHi holds significant potential to advance digital pathology by enabling optimized multimodal tissue analysis and supporting future developments in computational pathology and AI-driven diagnostics.

## Introduction

Classical histology involves the preparation of thin tissue sections suitable for microscopy examination. This entails embedding the tissue sample in paraffin and slicing it with a microtome. However, the sectioning process, in particular, irreversibly alters the tissue, and the stained sections provide inherently two-dimensional (2D) information. Furthermore, the examined tissue commonly represents only a minimal fraction of the entire sample. Recently, the multimodal fusion of histological imaging with three-dimensional (3D) scans of tissue has been proposed^1–3^ to enhance the analysis pipeline by combining complementary information from both imaging modalities. A key step in this fusion process is the alignment of corresponding structures between datasets, which can be done manually by visual inspection^4,5^ or using advanced image registration techniques^6^. In cases where 2D-to-3D matching is required, identifying corresponding sections can be challenging. This issue arises from potential misalignment between the sectioning axis and the 3D scan, which can lead to elastic deformation of the specimen. Addressing these issues may require virtual slicing of the 3D volume *in silico* and/or digitally correcting for deformations through transformations^5,7–9^. Furthermore, differences in image resolution between modalities can hinder an exact fusion of identical structures. The integration of Light Sheet Fluorescence Microscopy (LSFM) and histological imaging has proven to be a powerful tool for tissue analysis and validation^10,11^. Stained histological sections allow for detailed examination of glandular structures and architectural patterns, as well as the identification of cell types and tissue structures within 3D LSFM scans. By systematically positioning the tissue sections within the 3D *in silico* scan, the analysis is extended into three dimensions, enriching the spatial information available in the workflow^12^. Multimodal image registration is essential for effective image fusion and remains a key focus in medical image processing^1,13^. While various software-based registration solutions exist, they often require manual selection of corresponding image pairs^14^ or specialized technical expertise^15^. In particular, registration of fluorescence and (immuno-)histochemistry images enhances medical and biological research by combining cellular details with tissue morphology. Warpy^16^, an open-source registration tool that extends ImageJ, necessitates storing data as QuPath projects, which can pose a barrier for non-technical users. To simplify the alignment of 2D histological sections within a 3D volume, fiducial markers have been introduced as an automated method to pair and align corresponding images^7,17^. While these markers significantly improve registration accuracy and reduce computational requirements, they also have drawbacks. They may increase sample size, complicate embedding, or even damage tissue, posing challenges for seamless integration into existing analysis workflows. To address this challenge, we developed a novel software tool, LitSHi (**Li**ght **S**heet meets **Hi**stology), which facilitates the registration of both 3D LSFM and 2D histology imaging data. Our primary focus is the analysis of tissue cylinders extracted from FFPE samples, aimed at preclinical and translational research applications. LitSHi enables rapid and accurate fusion of these modalities, either through user-defined landmarks or through automated matching, with minimal user interaction. This approach improves upon traditional side-by-side comparisons of similar slices by enabling precise identification of corresponding structures and full 3D visualization. We demonstrate LitSHi’s capabilities by fusing LSFM and histology images of testicular tumor specimens, extending the analysis, for example, of intrinsic fluorescent basal membranes of seminiferous tubules into the third dimension. Using LitSHi, the automation of the formerly laborious and computationally intensive process of aligning LSFM and histology images has the potential to significantly enhance the efficiency of digital pathology, thereby contributing to ongoing research and clinical applications.

## Material and methods

### Workflow overview

**Fig. 1:**
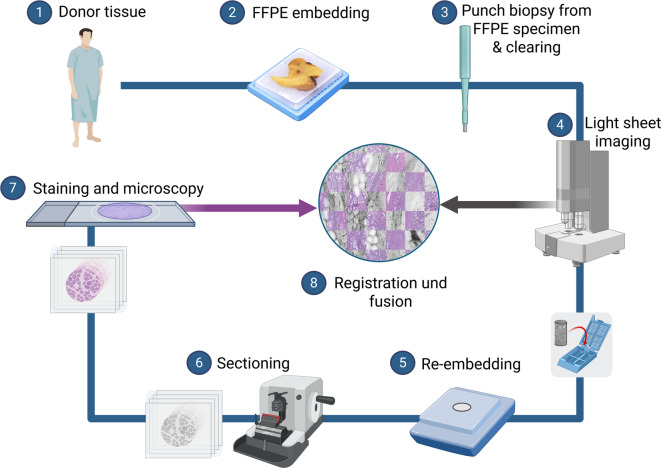
Overview of the complete workflow for tissue processing, imaging, and multimodal registration. (1–2) Archived patient biopsy samples from a previous study^11^ were fixed and embedded in paraffin. (3–4) Punch biopsies were extracted and cleared for light-sheet microscopy. (5–7) After imaging, tissue cylinders were re-embedded, sectioned, and stained for conventional microscopy. (8) The resulting light-sheet and histological images were registered and fused for multimodal analysis. The main contribution of this publication is highlighted by the purple and grey arrows. Created in BioRender. Viessmann, N. (2026) https://BioRender.com/s29xbrn.

For the development and evaluation of our novel alignment and registration approach, we used archived samples from patient biopsies collected in a previous study^11^. These samples were subsequently fixed and embedded in paraffin (see Fig. 1(1 and 2)). From the resulting blocks, punch biopsies were extracted and cleared for light-sheet imaging (see Fig. 1(3 and 4)). After imaging, the tissue cylinders were re-embedded, sectioned, and stained for microscopy (see Fig. 1(5 to 7)). This tissue processing pipeline has been established in previous publications^18–20^. Based on the acquired images, expanded the workflow through registering and fusing both modalities (see Fig. 1(8)). Fig. 1.

### Sample origin and preparation

Paraffin blocks (testicular tumor and pancreas specimen) were obtained from the Department of Pathology, University Medical Center (Goettingen, Germany). Pathological findings were made as part of each patient’s diagnostic process. Tissue cylinders with a 3 mm diameter were taken from the paraffin blocks using a punch biopsy needle (pfm medical GmbH, Germany). Punch biopsies that were not nuclear-stained were deparaffinized and rehydrated in a descending alcohol series (4 h xylol at 60 $^{\circ }$C, 59 h xylol, 2 $\times$ 12 h 100% ethanol, 2 $\times$ 4 h 96% ethanol, 2 $\times$ 4 h 75% ethanol, 4 h 60% ethanol, 4 h 50% ethanol, 4 h H$_{2}$O) before being embedded in gellan gum (Phytagel, Merck KGaA, Germany; 0.7% in H$_{2}$O) for stabilization. After a washing step in phosphate-buffered saline (PBS), the embedded samples were cleared by dehydration in an ascending alcohol series (4 h 30% ethanol, 16 h 50% ethanol, 4 h 70% ethanol, 4 h 90% ethanol, 16 h 100% ethanol, 4 h 100% ethanol) and placed in benzyl alcohol/benzyl benzoate (BABB, ratio 1:3) to adjust the refractive index, thereby reducing light scattering and absorption. Punch biopsies stained with a nuclear dye (TO-PRO$^{TM}$−3 Iodide, Thermo Fisher Scientific Inc., USA) were treated as described previously^11^. The protocol was adapted to use a TO-PRO$^{TM}$−3 concentration of 1:1000, and no counterstain with eosin was applied. Rather than embedding in Phytagel, these samples were stabilized in pre-cut, cleared blocks of gellan gum during measurement. Tissue clearing was performed at room temperature with gentle shaking until sufficient transparency was achieved, a process that could take up to five days.

### LSFM acquisition

Fluorescence microscopy was performed using the UltraMicroscope Blaze$^{TM}$ (Miltenyi Biotec B.V. & Co. KG, Germany). The cleared samples were attached to the sample holder and placed into a cuvette filled with ethyl cinnamate (ECI), used as a non-corrosive and non-toxic alternative to BABB with a comparable refractive index. Fluorescence excitation was provided by an NKT SuperK Extreme white light laser with 0.6 W optical output power in the visible spectrum (NKT Photonics A/S, Denmark). The Blaze$^{TM}$ is equipped with a 4.2-megapixel sCMOS camera with a 2,048 $\times$ 2,048 pixel resolution (Excelitas Technologies Corp., Germany). Standard measurement parameters included a numerical aperture of 0.163 and a light sheet thickness of 3.9 $\mu$m. The resolution in the XY-plane with the 4$\times$ magnification objective lens was 1.6 $\mu$m, with a Z-step size of 2 $\mu$m. The filter sets for excitation (ex) and emission (em) wavelengths, including an optical bandpass filter, were: 500 nm ex/20 nm & 535 nm em/30 nm; 560 nm ex/40 nm & 620 nm em/60 nm; and 710 nm ex/75 nm & 810 nm em/90 nm. Images were captured using ImSpector software (version 7.5.2, Miltenyi Biotec B.V. & Co. KG, Germany). LSFM data analysis was performed using Zeiss arivis Pro software (Carl Zeiss Microscopy GmbH, Germany).

### Embedding, sectioning, and staining protocol

After LSFM examinations, tissue samples were further processed for histological analysis by placing them in xylene for 1.5 hours, embedding in paraffin, and cutting into 2 $\mu$m sections. For staining, tissue slices were deparaffinized (60 $^{\circ }$C, 30 minutes) and rehydrated through a descending ethanol series. H&E staining was performed according to the manufacturer’s protocol. To detect immune cells within the tissue, immunohistochemistry targeting the transmembrane protein CD45 was performed. The rehydrated tissue slices were treated as follows: target retrieval solution (pH 9, 100 $^{\circ }$C, 20 min), H$_2$O$_2$ (10 min, room temperature), Seablock (20 min, room temperature), CD45 (rabbit anti-human, clone ERP20033, Abcam Limited, Cambridge, UK, 1:2000, 4 $^{\circ }$C, overnight), secondary antibody (anti-rabbit horseradish peroxidase, Histofine, Nichirei Biosciences Inc., Japan; 30 min, room temperature), and finally AEC (3-Amino-9-ethylcarbazole) substrate (20 min, room temperature). Intermediate wash steps were carried out with Tris-(2-amino-2-(hydroxymethyl)−1,3-propandiol) buffer (2 $\times$ 5 min, room temperature). Tissue slices were mounted with the corresponding medium and covered with a cover glass for microscopic evaluation.

### Microscopy

Each slide was digitized using a Laser Scanning Confocal Microscope 700 (Carl Zeiss Microscopy GmbH, Germany) with a resolution of 0.3 $\mu$m per pixel. All images were saved in TIFF format. Regions containing small artifacts, such as disruptive particles, were manually cleaned using graphic editing software, such as GIMP^21^.

### Software development

LitSHi was implemented in C++ using Visual Studio 2022 (version 17.11.4, Microsoft Inc.). Image processing was performed using open-source libraries, specifically OpenCV^22^ and ITK^23^, while image registration was conducted using elastix^24,25^. The graphical user interface was built with ImGui^26^. LitSHi, along with the required elastix parameter files, is available at^27^.

### Visualizations

The images shown in the figures were manually processed and assembled using graphic editing software such as GIMP^21^. All diagrams were created with draw.io^28^. The 3D rendering was generated using Zeiss arivis Pro software (Carl Zeiss Microscopy GmbH, Germany).

### Experimental hardware setup

The development and all performance tests in this study were executed on a machine with the following specifications. OS: Windows 10 Pro(x64); CPU: AMD Ryzen$^{TM}$ 9 7950X3D; RAM: 64 GB.

## Results

### Localization pipeline

**Fig. 2:**
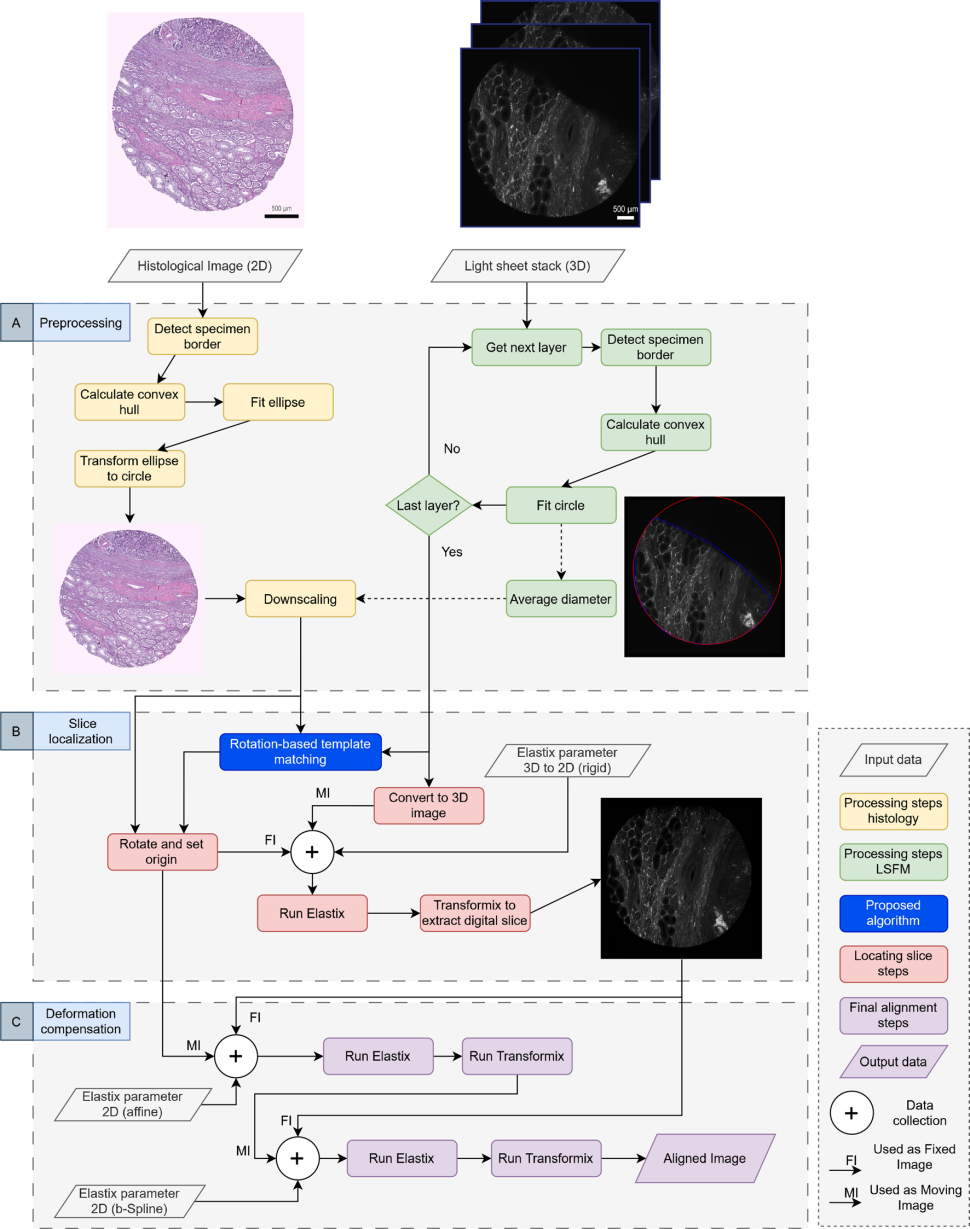
Workflow for matching histological sections to digital light sheet slices using LitSHi. The process is divided into three main phases for clarity. In (**A**), all input images are preprocessed to enable optimal alignment. In (**B**), the best-matching z-layer of the light sheet stack is identified using a rotation-based template matching algorithm. This estimated layer provides the initial reference for the 3D-to-2D image registration process, which iteratively refines the position of the digital cutting plane. Finally, in (**C**), the histological slice is aligned to the previously estimated digital slice through two cycles of 2D-to-2D image registration: first, an affine transformation adjusts the overall shape of the histological slice to the digital reference, followed by an elastic transformation that corrects for distortions introduced during tissue sectioning.

The developed workflow accepts both the 2D histological image and the 3D LSFM stack as input, with each modality processed through separate pipelines, indicated by different colors in Fig. 2A. LitSHi’s primary objective is the automated identification of a section within the LSFM stack that best matches the histological section digitized under a microscope. The histological image is assigned as the fixed image (FI), remaining unaltered during registration, while the LSFM image stack serves as the 3D moving image (MI). During sectioning, structural alterations may occur, often resulting in tissue deformation, where the typical circular shape of the specimen may appear stretched into an elliptical form in the 2D image. Since the sample is scanned before sectioning, its cylindrical morphology is preserved, so each layer, except for edge cases at the top and base, should display a circular cross-section. To facilitate accurate matching between modalities, the deformed histological image is first transformed into a uniform circular shape (see Fig. 2 A). A preliminary coarse search for a corresponding slice is then performed using template matching^29,30^ (see Fig. 2 B). Once a matching candidate is identified, both images undergo a 3D-to-2D rigid alignment, followed by affine and elastic registration for further refinement (see Fig. 2 C). A dedicated coarse detection step for the initial placement of the slice in the volume is crucial for precise and computationally optimized alignment.

### Overview of the LitSHi registration tool

**Fig. 3:**
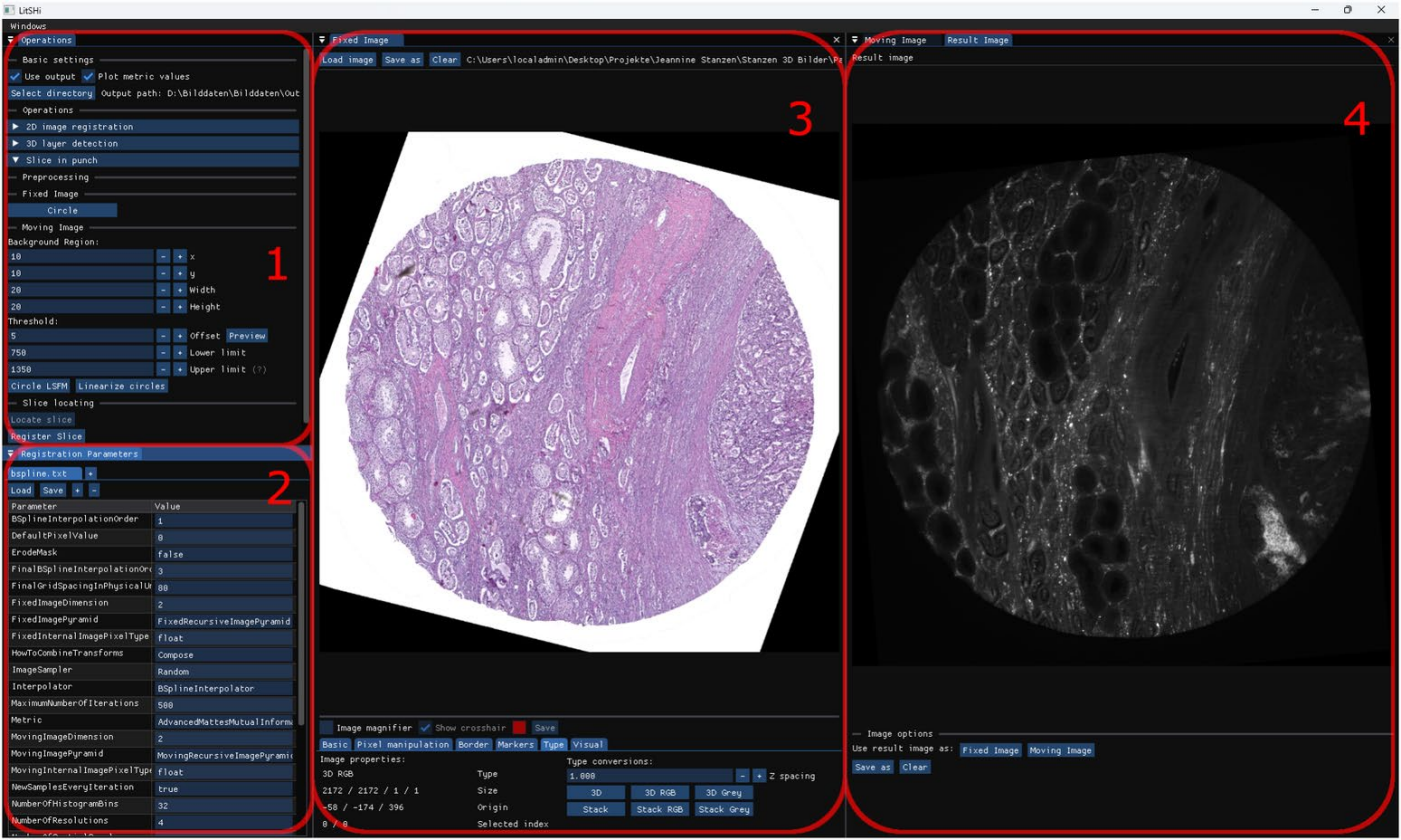
Screenshot of the LitSHi GUI for an exemplary workflow, with four frames highlighted by red borders for clarity. Frame 1 shows the functions window, which allows control of the individual algorithms provided by LitSHi. Frame 2 displays the parameter window for the elastix image registrations. Frame 3 contains the input window for the fixed image, and Frame 4 presents the result window to display the transformation results.

LitSHi was designed as an easy-to-use alignment and registration tool intended for both biologists and technical staff. It accepts TIFF stacks for 3D volumes and commonly used image formats such as.png and.jpg for 2D sections. Through the use of OpenCV, it can handle all common bit depths and color spaces. The internally used depth per channel is 8 bits; therefore, images of other bit depths are automatically converted during the loading process. LitSHi combines common image registration tasks such as 2D-to-2D transformations and is also suitable for users who are not familiar with computer science or software development. Due to the capability of loading elastix parameter files, editing individual parameters, and saving the files again, one can easily test different registration settings and determine the best configuration for the current task. Additionally, our software tool offers an optional plot of metric values after each registration step to help fine-tune the registration parameters. Furthermore, it can also be used to visually analyze transformation results, for example by generating a checkerboard image. When it comes to the task of 3D-to-2D image registration, LitSHi offers two different approaches. The first is based on manually set marker points, which are used to determine a starting location for subsequent intensity-based image registration steps. The second is the algorithm presented in this study, which can automate the process of finding the initial alignment for tissue punches. The main GUI of LitSHi is shown in Fig. 3. To fuse a histological slice with an LSFM stack from a tissue punch, following the pipeline illustrated in Fig. 2, the required image data are first loaded into the *Fixed Image* and *Moving Image* windows (Fig. 3, frames 3 and 4). All functionalities associated with *(A) Preprocessing* and *(B) Slice localization* are accessible via the collapsing header *Slice in punch* (frame 1), which also activates the rotation-based algorithm used for slice localization. Parameter files required for all elastix-based registration steps must be provided in the parameter file window in frame 2. After successful slice localization, the registration problem reduces to a 2D-to-2D transformation; consequently, *(C) Deformation compensation* is conducted under the collapsing header *2D image registration*, which serves as the general interface for all subsequent 2D-to-2D image registration procedures.

### Preprocessing

**Fig. 4:**
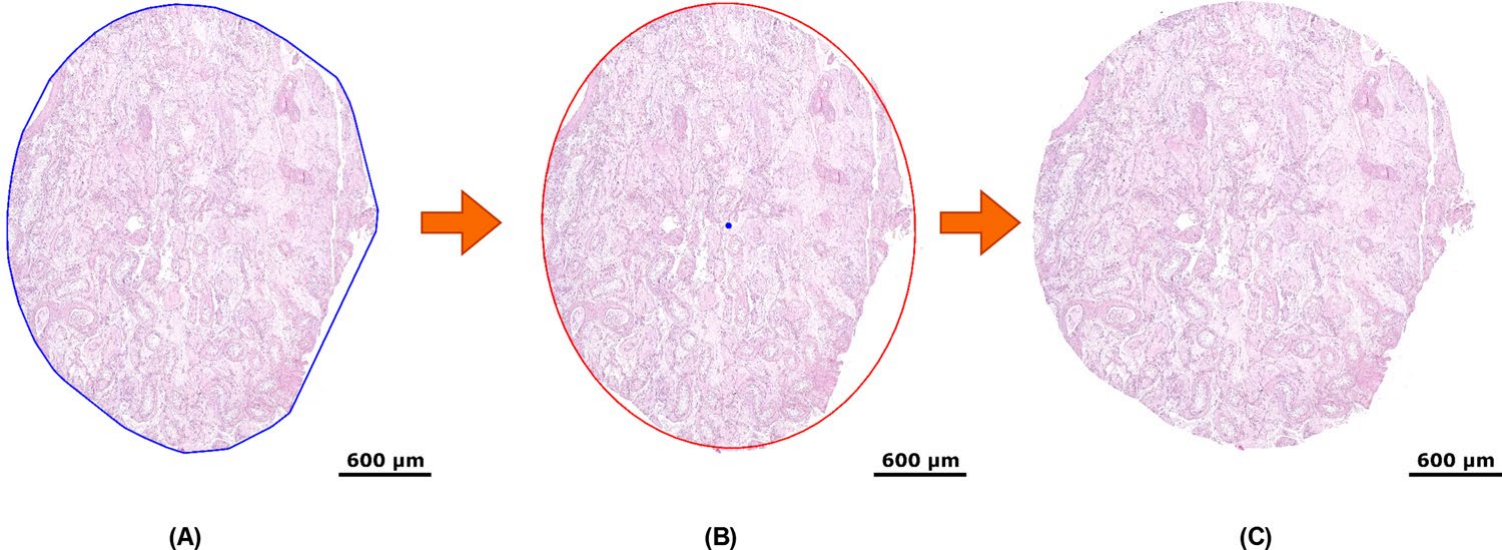
Transformation steps of the histological image. (**A**) shows the unaltered specimen with the calculated convex hull depicted as a blue polygon. (**B**) illustrates the fitted ellipse based on the convex hull. The center of the ellipse is marked by a blue point, which is later used for the centered alignment of the histological image with the individual LSFM layers. Finally, (**C**) depicts the outcome after affine transformation.

This processing step prepares the input images and computes essential information for subsequent slice localization. The primary goal is to transform the histological section back into a circular shape, as the tissue, which is extracted from the specimen prior to imaging, can be approximated *in silico* as nearly circular. In contrast, the histological image may deform into a more elliptical shape due to shearing during sectioning. To achieve this, the contour (circumference) of the sample is first detected, and based on these marked pixels, a corresponding convex hull is computed^31^. The convex hull provides a robust basis for estimating the approximate shape of the section, even in the presence of shearing or other deformation artifacts. As shown in the example in Fig. 4A, the convex hull is first computed from the tissue contour. An ellipse is then fitted within this convex hull (Fig. 4B), and its center is calculated. Unlike the convex hull–which would yield the center of gravity–the fitted ellipse produces a center point that is directly comparable to the centers of the circular images in the light-sheet stack. To morph the histological section into a circle, the fitted ellipse is geometrically transformed. The required circular shape was achieved by elongating the images orthogonally to the major axis. As a result, the diameter of the resulting circle equals the length of the elliptical major axis. The center and diameter of the resulting circle are then used in the next step to align and scale the histological image relative to the LSFM layers. An example of a rectified histological image is depicted in Fig. 4C.

The preprocessing of the LSFM stack entails computation of contours and centers for each slice individually, following the green workflow path depicted in Fig. 2A. Analysis of the upper and lower borders of the *in silico* volume can be particularly challenging due to the uneven specimen surface or initial cuts that are not orthogonal to the LSFM captured layers. In such regions, circular contour detection is often unreliable; therefore, only well-defined circular or distinctly semicircular shapes validated by the number of points arranged in a circular pattern, are retained for subsequent steps, while ambiguous layers are discarded. To optimize the detection in these particular scenarios, the points of the convex hull are filtered using RANSAC^32^. Given that the histological image has a higher resolution than the LSFM images, the diameters of all valid layers are quantified and averaged to derive a representative scaling factor, which is also robust to random variations inherent to RANSAC. This average diameter is subsequently used to rescale the histological section to match the spatial dimensions of the LSFM data.

### Slice localization

Following preprocessing, the histological images were successfully downscaled, allowing both modalities to be directly compared in the similarity analysis. Despite the challenge that a 2D plane can move with six degrees of freedom within a 3D volume, LitSHi achieved reliable alignment through an automated template matching procedure that leveraged the circular geometry of both the histological and LSFM images. This approach facilitates the identification of an initial seed point for 2D-to-3D image registration, thereby enabling a more efficient and accurate alignment. The following subchapters provide detailed descriptions of these two key steps.

#### Template matching

**Fig. 5:**
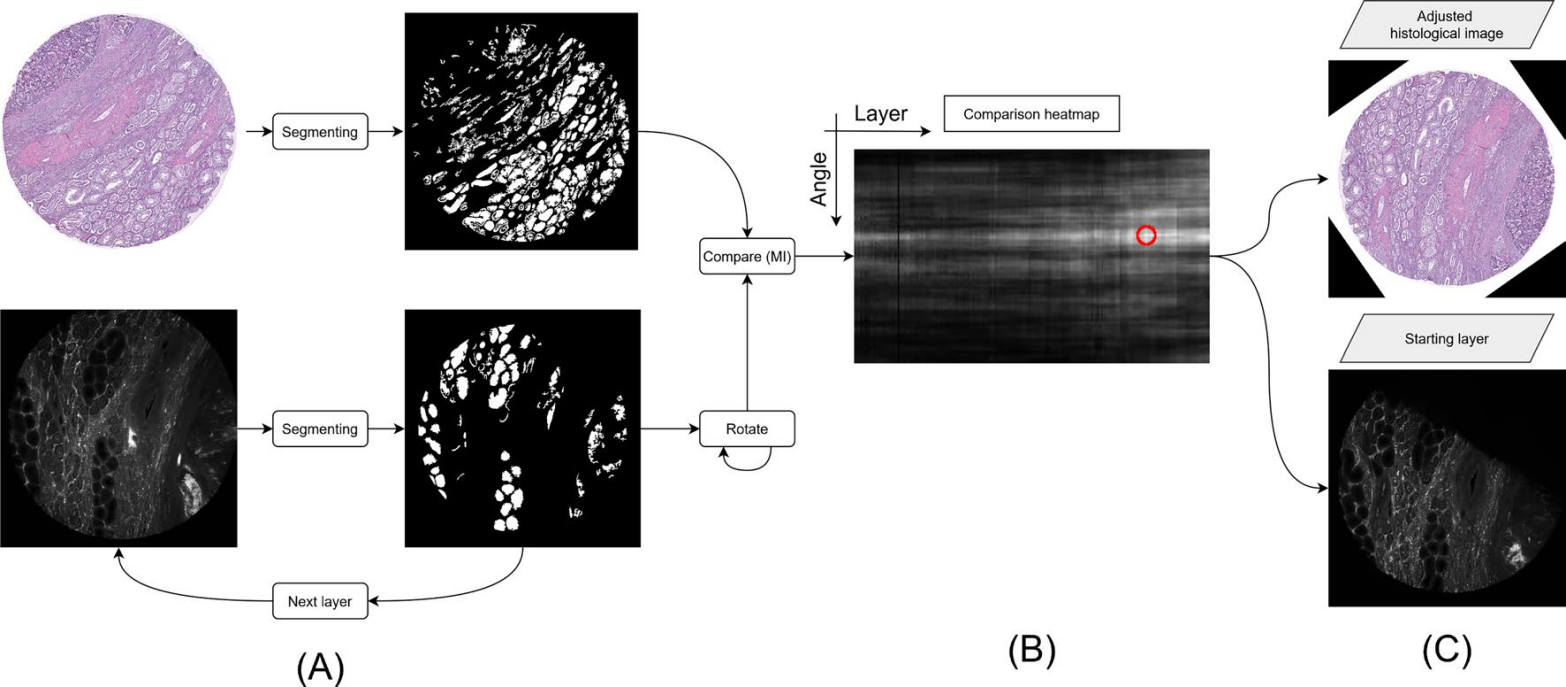
Workflow and result of the rotation-based template matching algorithm. The histological image is compared to each layer of the LSFM stack. Since the input images are from different modalities, they cannot be directly compared. Therefore, both images are initially segmented to detect clearly distinguishable tissue features. To approximate the optimal rotation for alignment, each layer segmentation is iteratively rotated during the comparison process (**A**). The algorithm generates a heatmap (**B**) that records all comparison results based on the Mattes Mutual Information metric, with the x-axis representing the layer number and the y-axis the rotation angle. The highest metric value indicates the best-matching layer and angle (marked with a red circle). In (**C**), for subsequent processing, the histological image – rather than the LSFM stack – is rotated, and its 3D origin adjusted to align with the best-matching layer.

Template matching is a well-established image processing technique^29,30^ that is commonly used to locate a subimage or template within a larger image. This approach utilizes a sliding window technique in which a template is systematically translated across the larger image. At each position, pixel-wise comparisons are performed between the template and the corresponding window region to compute a similarity metric. In the context of this work, the 2D histological image functions as the template that is matched against the 3D *in silico* volume. Instead of moving the template in the x- and y-directions within the plane, the template is rotated about the z-axis in 2$^{\circ }$ increments for each image in the stack. The step size was experimentally determined. Smaller angles did not show any improvement. This coarse alignment is further refined in the later processing steps. In the context of LitSHi, the objective of the implemented algorithm is to facilitate precise alignment of corresponding substructures, thereby ensuring optimal congruence between images. These structures are primarily represented by clearly distinguishable features, such as those highlighted by white borders (e.g., basal membranes of seminiferous tubules). Segmentation of these structures was achieved through a combination of thresholding and morphological closing, followed by filling any remaining holes within the objects. Additionally, the objects were filtered based on their respective sizes to prevent the accumulation of adjacent structures. Both the histological image and the LSFM stack were processed using the same segmentation pipeline, with dedicated parameterization for each modality. Following the segmentation process, the 2D image (the template) is matched with the first valid plane that depicts a complete circle within the 3D stack. Since the orientation of the section may differ from the *in silico* plane, the latter is rotated with bilinear interpolation, and a similarity score is computed for each candidate. In this context, Mattes mutual information^33^ was selected as the metric, as it has been demonstrated to be highly effective for multimodal data^34,35^. This approach is advantageous because it remains robust to missing structures that may be absent in one imaging modality, relying instead on the overall statistical dependency between the two images. The implemented template matching algorithm is illustrated in Fig. 5.

#### 3D-to-2D image registration

Using 2D template matching, a coarse estimation of the position of the histological section within the LSFM stack is established. This position is then used as the starting point for a 3D-to-2D image registration, where the entire stack is treated as a 3D ITK^36^ image. During this process, the position of the stack is adjusted rigidly, allowing only translational or rotational transformations. For each iteration, the advanced Mattes mutual information is computed for the resulting overlay. In this context the stack is designated as the MI, while the histological section is defined as the FI. This iterative process was repeated for 300 iterations, after which the optimal alignment was determined. The number of iterations was heuristically estimated, balancing accuracy and computational time. However, this initial match may still require further refinement to account for elastic deformations present in the histological slice.

### Deformation compensation

**Fig. 6:**
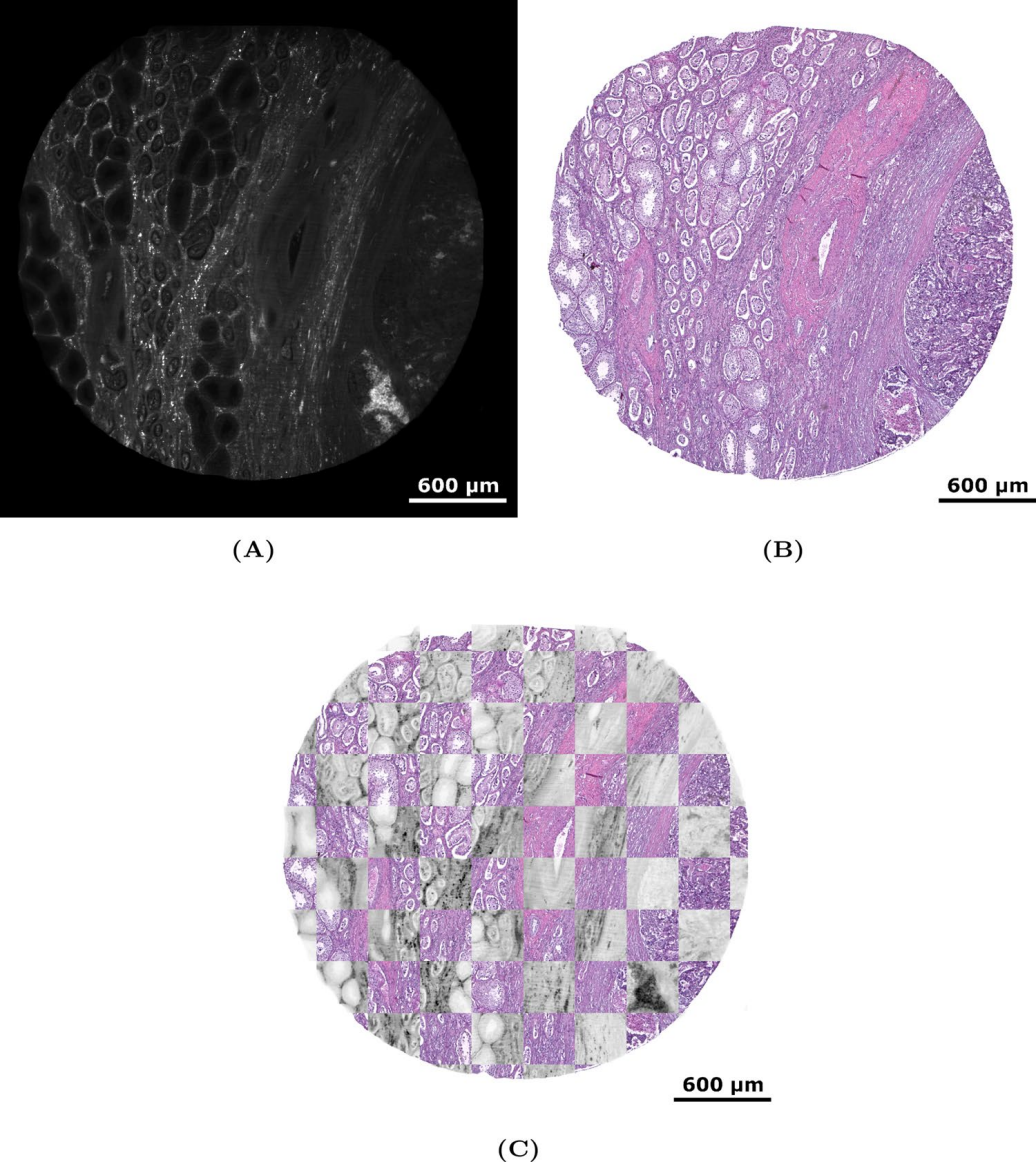
Registration results. (**A**) shows the determined digital cutting plane in the LSFM stack. The elastically transformed histological image is depicted in (**B**). For a visual comparison, both (**A**) and (**B**) are used to create a checkerboard pattern in (**C**), where the color of the digital slice is inverted and its contrast is enhanced.

Following the rigid alignment of both the *in silico* plane and the histological section, a two-fold fine alignment registration process is pursued, with increasing degrees of freedom. First, both modalities are registered using an affine approach. At this stage, the extracted plane is treated as the ground truth (FI), since the sample has not been cut, and its geometrical integrity remains intact. The number of iterations is again set to 300. Once the ideal transformation that yields the highest mutual information is identified, the histological slice is adjusted accordingly. For the second fine alignment registration, the transformed section is once more designated as the MI in a B-spline-based alignment. This process significantly increases the degrees of freedom, necessitating a greater number of iterations (2000 over 4 resolutions) to achieve optimal alignment. Fig 6 shows an example of the final results.

### Comparison between the proposed and previous workflows

#### Comparison to manual patch alignment

**Fig. 7:**
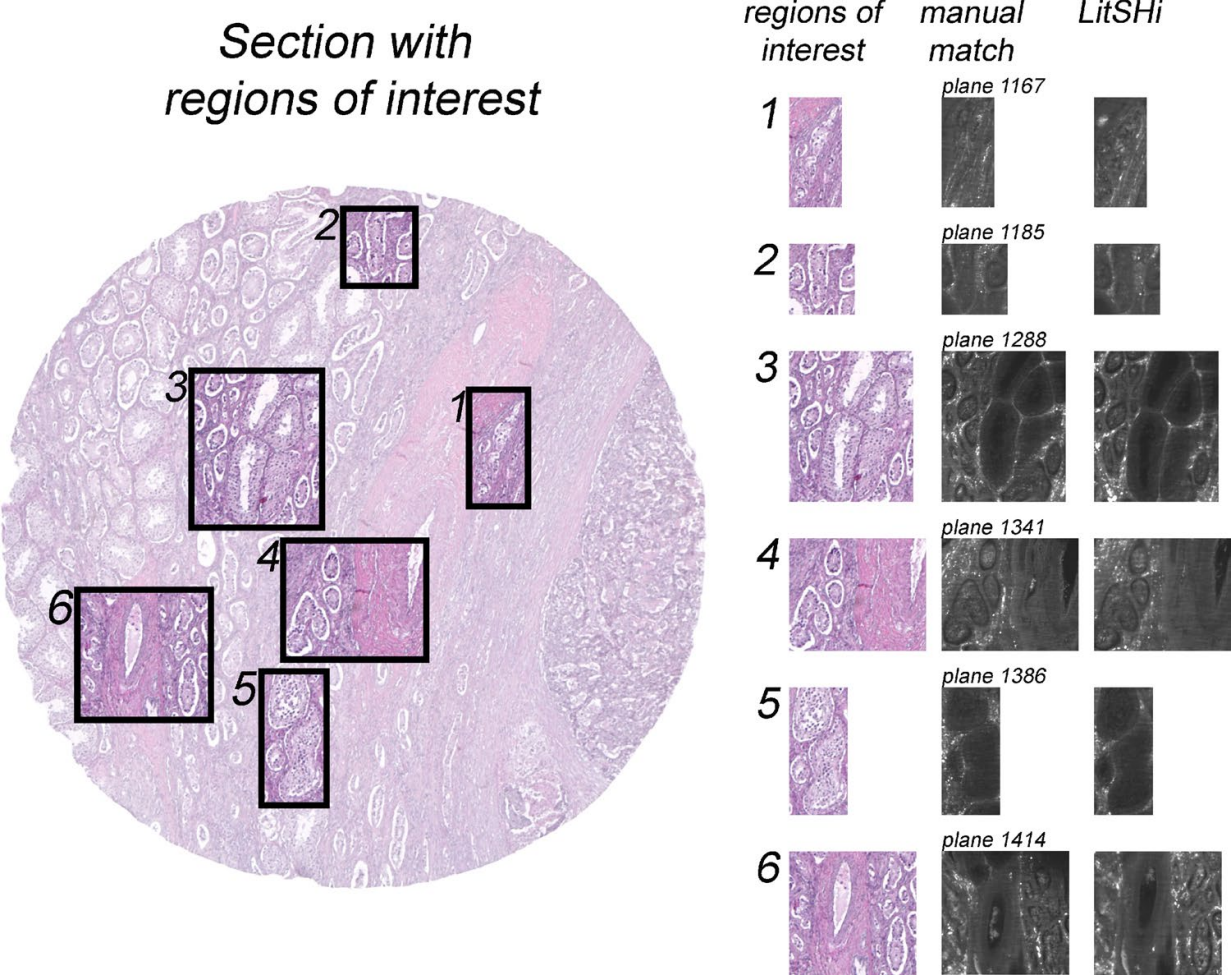
Qualitative comparison of both manually and automatically matched regions of interest (1–6) using LitSHi. While expert-based identification of corresponding histology and LSFM patches yielded adequate pairings, it required manually navigating through the image stack. Due to the skewed sectioning, the histological section is positioned between *in silico* planes 1167 and 1414. Thus, while predominantly large structures appear to be visually similar the corresponding plane cannot be precisely identified by manual stepping through the z-stack. With LitSHi, this process is significantly streamlined.

In previous attempts to match LSFM planes with histological slices, the correspondence was determined manually by traversing the 3D image stack to identify similar regions^11^. However, this manual process is time-consuming, error-prone, and imprecise. In this study, we compared the expert-identified matches with the results produced by LitSHi. To enable a fair comparison between expert annotations and LitSHi’s performance, the histological image was transformed into a circular representation (as shown in Fig. 4) and then rigidly registered to the LSFM planes selected by the expert. The comparison between manually selected LSFM stack ROIs and automatically aligned sections is shown in Fig. 7.

A rendering of the registered section within the 3D volume is shown in Supplementary Figure S2, highlighting the skewed orientation of the plane inside the volume. While the coarse similarity between the manually selected and automatically registered sections and the histological image is apparent, the morphology of finer structures shows noticeable deviations. To quantitatively assess these differences, we computed the absolute values of the Normalized Cross-Correlation (NCC). Since NCC values can be negative when local intensity patterns are inverted, using absolute values provides a more intuitive measure of structural similarity independent of contrast polarity. The results are summarized in Table 1.

**Table 1:** Absolute NCC values for manual and LitSHi alignments across patches.

Patches from Fig. 5	Manually selected patch	Patch generated by LitSHi
Patch 1	0.05	0.16
Patch 2	0.04	0.34
Patch 3	0.20	0.38
Patch 4	0.20	0.32
Patch 5	0.17	0.41
Patch 6	0.10	0.34
**Mean across patches**	**0.13±0.07**	**0.33±0.08**

In our experiments, the expert required approximately 40 minutes to manually annotate and match corresponding patches between the two imaging modalities. This process involved carefully traversing the image stack to identify and align regions of interest. In contrast, LitSHi significantly streamlines the task by automating the matching process with substantially improved efficiency. LitSHi completes the computation in just a fraction of the time, reducing the required effort to about one-eighth of the manual matching time. For the same stack and histological image given to the expert the processing time equaled about 5 minutes, where 2 minutes accounted for the registration and translation performed by elastix.

#### Comparison to manually defined landmark-based pre-alignment

While the performance comparison on the patches showed an improvement over manual identification of corresponding regions, the main advantage of LitSHi, namely, identifying an initial starting position for 2D-to-3D registration, was not fully explored. In order to compare our rotation-based template matching approach with methods for determining an initial position for 2D-to-3D registration, we added a landmark-based alignment feature to LitSHi. This feature enables manual annotation of regions within the 3D *in silico* data that resemble structures in the histological image, a commonly used approach for registration problems as for example implemented in Zeiss arivis. For our experiment, we used four different LSFM stacks - three stacks of testicular and one stack of pancreatic tissue - and 21 histological sections. For each section, three manually selected marker points were annotated both within the corresponding LSFM stack and on the section itself. Based on these points, the corresponding planes were extracted and matched to the individual histological sections using rigid, affine, and elastic registration steps as described in the previous subsections. We then evaluated the similarity of the landmark-based and rotation-based initialization methods by calculating the absolute NCC score for the resulting planes and five histological sections. The results are shown in Table 2.

**Table 2:** Comparison of the similarity achieved by landmark- and rotation-based template matching initialization for 21 corresponding histological sections and LSFM planes.

Initialization strategy	Mean absolute NCC score ± standard deviation
landmark-based	0.20±0.12
Rotation-based template matching	0.21±0.12

**Fig. 8:**
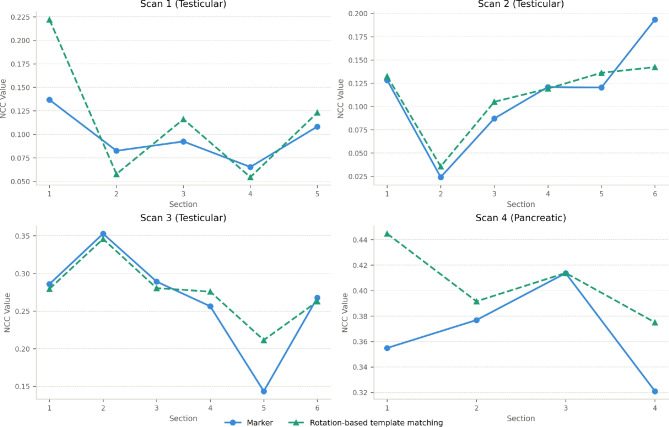
Per-section absolute NCC scores for landmark-based (Marker) and rotation-based template matching initialization across all four LSFM stacks (Scans 1–3: testicular tissue; Scan 4: pancreatic tissue). Each data point represents the NCC score achieved for an individual histological section. While both strategies yield comparable mean performance, section-level profiles reveal notable variability within individual scans, reflecting differences in local tissue heterogeneity, sectioning-induced deformation, and the degree of structural overlap between modalities.

Although the mean NCC scores in Table 2 suggest comparable overall performance between the two initialization strategies, aggregated statistics alone can obscure meaningful per-section variability. In multimodal registration, NCC values are inherently influenced by local tissue heterogeneity, stain intensity, and the degree of structural overlap between the histological section and the corresponding *in silico* plane – factors that vary considerably between individual sections even within the same scan. To provide a more nuanced view of alignment quality, Fig. 8 shows NCC scores per-section for both initialization strategies across all four LSFM stacks.

The profiles reveal that neither method consistently outperforms the other across all sections: in some cases the rotation-based template matching yields higher NCC values, while in others the landmark-based approach performs comparably or better. Taken together, these results suggest that the two initialization strategies are broadly equivalent in terms of registration quality, while rotation-based template matching eliminates the need for time-consuming manual landmark annotation.

As an additional experiment, we also evaluated the use of SIFT^37^ feature descriptors to align both modalities. We computed the mean distance between corresponding feature points and inverted this value to allow direct comparison with the MI metric used in our rotation-based template-matching approach. As shown in Supplementary S1, a maximum in the inverted $L^2$ metric was observed, however, inspection of the resulting matches revealed no substantial correspondence.

### Correlative imaging enables multimodal investigation of malignant cells

**Fig. 9:**
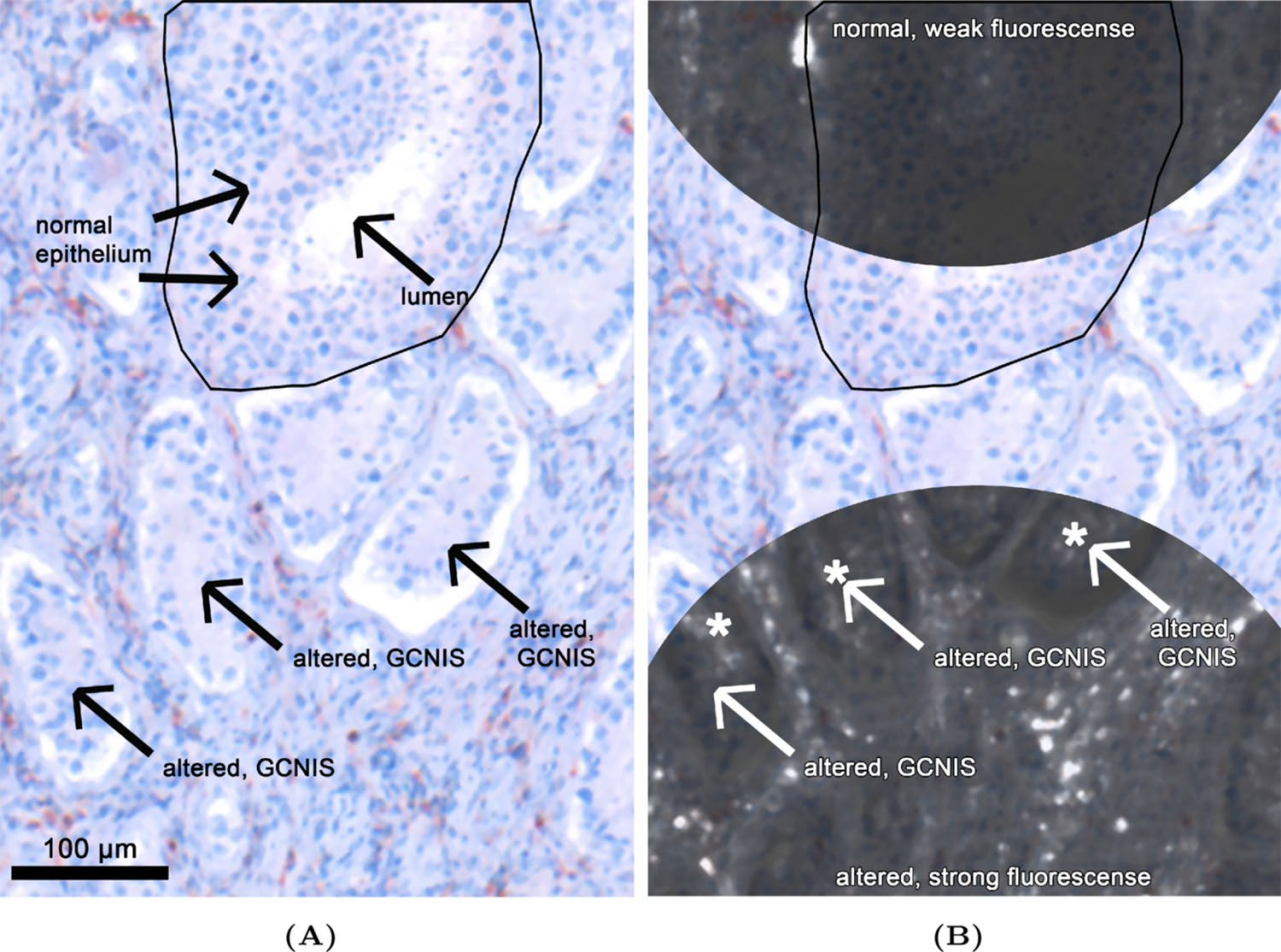
Analysis of the fused image data. (**A**) displays a section of the elastically transformed tissue slice, stained with an anti-CD45 antibody. The grayscale regions correspond to the identified *in silico* cutting plane shown in (**B**). The dataset includes unaltered seminiferous tubules (black frames) with well-defined lumina (arrows) and weak fluorescence in the germinal epithelium. In contrast, the pathologically altered tubules (arrows) lack lumina, and the nuclei of the few remaining germ cells appear enlarged and hyperchromatic, exhibiting intrinsic fluorescence signals (*). The corresponding histological tissue slice confirms the presence of the germinal epithelium and the altered seminiferous tubules, most likely displaying germ cell neoplasia *in situ* (GCNIS) morphology.

The utilization of LSFM imaging facilitates a comprehensive analysis of whole testicular tumor biopsies, thereby optimizing the spatial visualization of both morphological and anatomical details. Building upon prior research^11^, our innovative registration-based pipeline facilitates a direct comparison of histology and LSFM and enables precise alignment, thereby enriching the analysis through multimodal fusion. Integrating histological section registration into LSFM imaging enhances the accuracy of identifying key diagnostic features within tumor samples. In a punch biopsy of a testicular germ cell tumor, light microscopic analysis revealed both unchanged testicular tubules with a regular germinal epithelium stratification and intact lumina, as well as tubules with an altered morphology of the germinal epithelium. These pathologically altered tubules lacked lumina, and the nuclei of the few remaining germ cells appeared enlarged and hyperchromatic. Such changes indicate a germ cell neoplasia in situ (GCNIS), a condition with critical diagnostic and prognostic implications. In the corresponding LSFM datasets, regions of the germinal epithelium that remain unaffected and healthy could be clearly distinguished from those exhibiting GCNIS-related alterations. While normal testicular tubules exhibited minimal fluorescence apart from the surrounding basement membrane–appearing unfilled and dark– tubules with GCNIS characteristics displayed strong fluorescence of germ cell nuclei, which densely filled the entire lumen. The tomographic LSFM dataset enabled the assessment of the spatial extent of GCNIS within the entire biopsy punch. Fig. 9 presents a fused histological section with the corresponding LSFM plane, extracted using LitSHi.

Elements such as tumor infiltration, germ cell neoplasia in situ, cellular pleomorphism or immune cell infiltration can now be examined in a 3D framework while maintaining direct reference to histological and immunohistochemical markers.

### Additional experiments and limitations

**Fig. 10:**
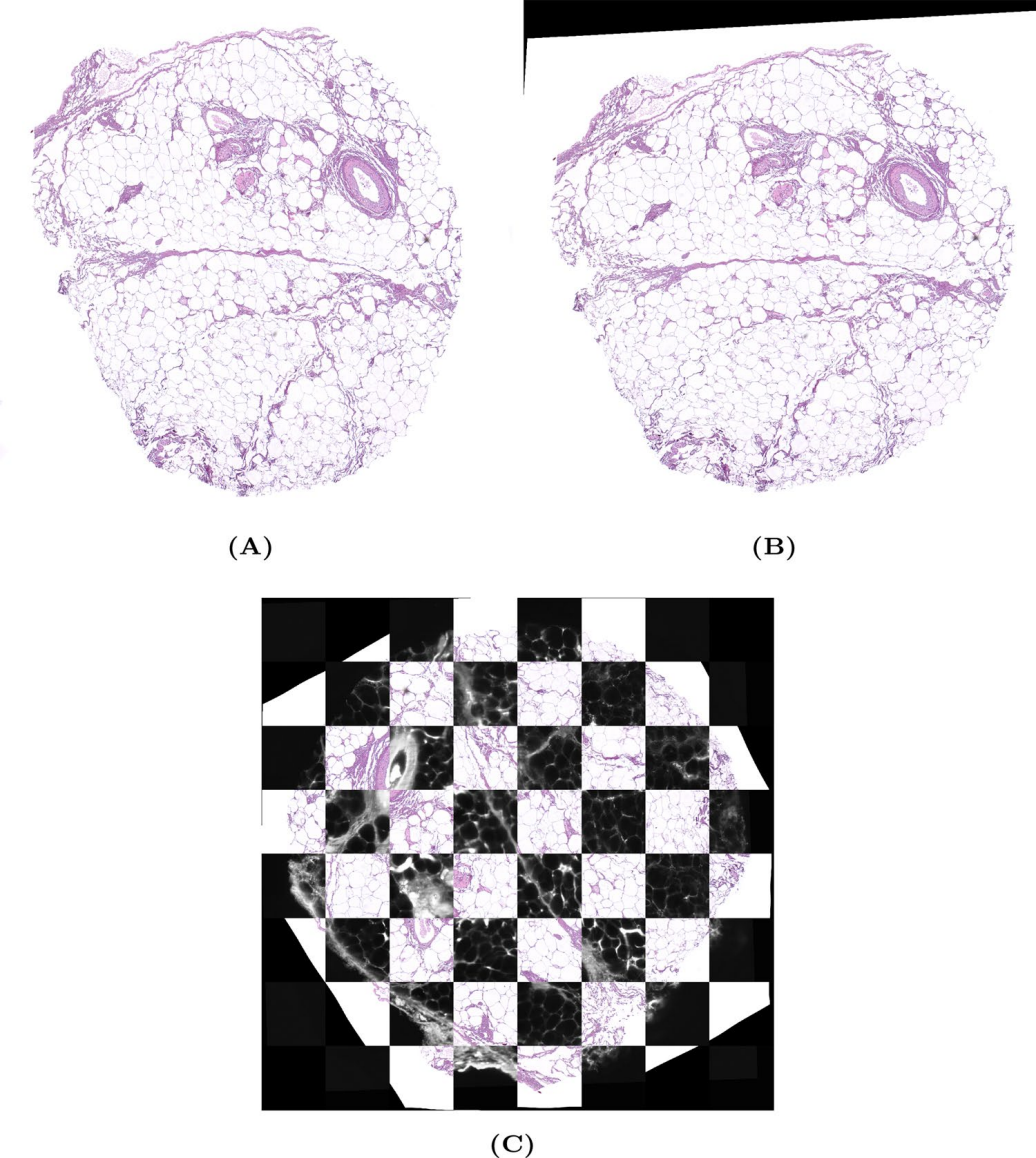
Processing pancreas-associated adipose tissue with minimal deformation using the LitSHi rotational template matching pipeline. (**A**) shows a deformed section prior to circularization. (**B**) presents the resulting circularized histology, which is subsequently aligned with the corresponding LSFM layer in (**C**).

To further investigate the applicability of LitSHi for multimodal fusion tasks, we additionally performed alignment and registration on pancreatic tissue biopsies. In these samples, the histological sections frequently underwent visible deformation during processing, resulting in non-circular shapes. Fig. 10 illustrates an example of a section whose deviation from circularity does not impair LitSHi’s performance.

**Fig. 11:**
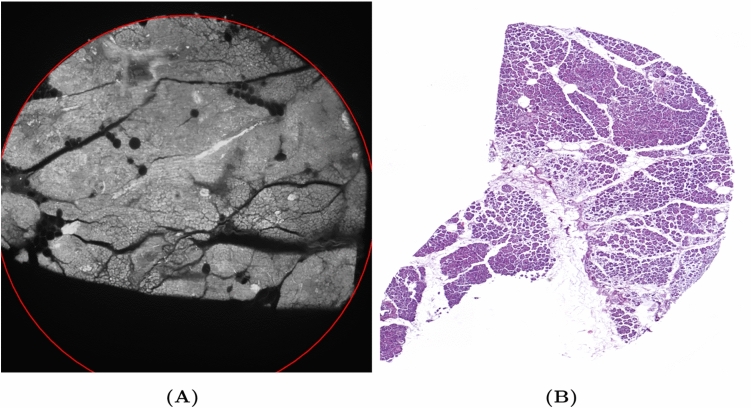
Failed circularization due to a severely deformed tissue section. (**A**) shows the detected circle for the LSFM plane, while (**B**) depicts the partially circularized histological section.

In contrast, when the histological section is severely distorted or portions of the punch biopsy are missing, LitSHi fails to generate an adequate starting layer and, consequently, cannot correctly align the modalities. In such cases, circularization breaks down, and the matched LSFM plane does not correspond to the correct anatomical location. Fig. 11 shows an example of this failure mode.

## Discussion

Our innovative registration-based tool, LitSHi, enables automated and precise alignment of 3D LSFM and 2D histology images of tissue cylinders extracted from FFPE specimens. In a multi-step process, the histological slice is preprocessed and coarsely aligned with an LSFM plane through rotation-based template matching. The position of the histological slice within the *in silico* volume is determined by measuring the similarity between the slice and the corresponding plane in the stack. To improve alignment quality, rotation is taken into account by generating multiple representations of each plane, each rotated slightly around the central z-axis. All generated candidates are then compared to the original histological slice, and an initial position within the stack is determined based on the highest computed similarity. After this initial alignment, the match is further refined through established rigid, affine, and elastic registration steps. The introduction of LitSHi has further refined our earlier work^11^ by automating the alignment of both modalities, thereby significantly improving precision. Previously, regions of interest spanning multiple planes could not be observed within a single LSFM image. Structures nearly 300 slices apart in the stack were challenging to analyze simultaneously–this limitation has now been eliminated, as all relevant image content is combined into a single plane. Overall, when compared to the manually selected patches, the LitSHi-generated alignment performed better by a factor of about 2.5, considering the mean across all patches (see Table. 1). However, since the NCC score was generally observed to be higher in larger patches, the performance cannot be overall generalized. Furthermore, we compared the performance of the rotation-based template matching initialization to the manual definition of a preliminary positioning of the starting plane based on landmarks. Here, we found that the automated template matching approach performs on par with the landmark initialization but requires no time-consuming definition of landmarks by the user (see Table 2). The integration of complementary LSFM structural data with highly specific histological stainings has been significantly accelerated–achieving an approximately eightfold improvement in efficiency compared to the previously labor-intensive manual process. This advancement supports the integration of correlative imaging into standard histological workflows and may contribute to more precise diagnosis and improved treatment planning, but further validation is still needed to confirm its diagnostic value. The combination of light microscopy with tomographic LSFM datasets from the same sample has been demonstrated to obtain complementary information. The distinct fluorescence characteristics of healthy and GCNIS cells within the germinal epithelium enable clear diagnosis in the LSFM dataset, facilitating spatial assessment of GCNIS across the entire biopsy specimen. The ability to evaluate discrete neoplasms and their precursors plays a crucial role in diagnosis, prognosis, and treatment planning for patients. By introducing LitSHi, we not only offer a user-friendly graphical interface for common image registration tasks but also simplified the identification of corresponding images. Consequently, manual identification of image pairs, a step still required by other solutions such as Warpy^17^ or MMIR^14^, is no longer necessary. Here, the quality of the registration process is highly dependent on the similarity between the moving and fixed images, which can be achieved if no additional alterations are made to the image sample. In cases where the tissue is sectioned after imaging, defining corresponding pairs becomes significantly more challenging. One potential solution to this challenge lies in the incorporation of fiducial markers, a concept proposed by^7^ and^17^. These markers, identifiable across both modalities, function as guides for precise 2D-to-3D alignment. In this case, no additional markers or references were necessary, as LitSHi utilizes the geometrical shape of the punch-hole biopsy samples. Here, each layer is represented as a circle with a defined center point, which can be easily detected in both modalities, thereby facilitating simplified alignment. While LitSHi uses elastix, it is a standalone tool for multimodal image registration and does not require any additional image processing tools such as ImageJ^38^, in contrast to Warpy^16^. Our repository includes the adapted Elastix parameter files used in our experiments, which can be+ easily loaded by LitSHi, thereby simplifying its use for non-technical users. Although stand-alone software solutions such as the Medical Imaging Interaction Toolkit (MITK)^39^ support multimodal image registration, they typically depend on manual user input, which not only requires specialized training but also makes the process time-intensive compared to our fully automated approach. Despite the notable advantages demonstrated by LitSHi and its workflow for fusing multimodal data, certain limitations persist. For now, LitSHi can only process 3D data as separate TIFF images, as provided by the light sheet microscope. For 2D histological images, the range of supported formats is currently limited to common standards like.png or.jpeg. More specialized file types, such as DICOM or.czi, which are supported by tools like Warpy^16^ due to its ImageJ^38^ origin, are not supported. However, this issue can be addressed through file conversion. In the future, we aim to support a broader range of image data types. Our algorithm performs a downsampling of the microscopic image to match the resolution of the LSFM stack; therefore, images with higher fidelity do not improve the overall alignment process. For the experiments, we used the equipment and data from our previous work^11^; however, the methodology is applicable to other imaging devices such as slide scanners, given the aforementioned restrictions on resolution. The size and resolution of the 3D stack and histological image are limited by the available RAM, which was sufficient enough for our experiments. The algorithm was developed specifically for the analysis of punch biopsies of FFPE-embedded testicular cancer tissue, but it also performed well on circular pancreatic specimen. While slight deviations from circularity do not impede circularization, the section must not diverge too substantially from the assumed shape. In such edge cases, incorporating alternative features, such as texture, could improve the alignment. The similarity measurement between images depends heavily on accurate segmentation of cell morphology, a limitation that currently restricts its application to this specific type of data. For now the thresholding is limited by manual definition, adaptation to different tissue types, and thus can lead to time consuming manual optimization or failure. This manifests itself as a drawback of the proposed methodology, where manual landmark-based registration might be more effective. However, due to LitSHi’s modular design, the software can be seamlessly extended with custom segmentation workflows or machine learning approaches, such as SAM^40,41^, thereby ensuring its adaptability to diverse research needs. This could enhance the applicability of LitSHi across a broader range of tissue types, reducing the need for future adaptations of the segmentation procedure. Additionally, minor design improvements are planned to enhance the stability of punch biopsy detection within each layer of the LSFM stack. Currently, this detection is achieved using RANSAC^32^, which produces varying results due to its stochastic nature. A deterministic, custom approach could replace RANSAC, thereby improving the algorithms robustness. In the future, we intend to integrate LitSHi into our histological workflow. One potential application is the fusion of a subset of H&E-stained slices from different locations within the scan. The most informative slices could be identified by leveraging complementary information from both modalities based on these fused images. Adjacent slices may then be selectively stained with more specific and, in most cases, more expensive dyes. In this context, LitSHi can serve as a verification tool for a targeted staining approach. Additionally, the generation of multimodal datasets based on the performed image registrations is planned. These datasets could play an integral role in developing new machine learning models, enabling the digital staining of non-specific LSFM scans.

## Conclusion

Our novel registration-based tool, LitSHi, facilitates the fusion of LSFM and histological images. This approach has been shown to enhance both the speed and precision of analysis compared to manual methods, thus broadening the scope of pathological tissue sample analysis. The automation streamlines the integration of complementary imaging modalities and supports the creation of multimodal datasets. From a forward-looking perspective, the method could be further optimized, with the resulting matched data potentially contributing to the development of multimodal machine learning applications, such as digital staining.

## Supplementary Information

Supplementary Information.

## Data Availability

The datasets used and/or analyzed during the current study are available from the corresponding author on reasonable request.

## Abbreviations

2D: Two-dimensional
3D: Three-dimensional
LSFM: Light Sheet Fluorescence Microscopy
LitSHi: Light Sheet meets Histology
PBS: Phosphate-buffered saline
BABB: Benzyl alcohol/benzyl benzoate
ECI: Ethyl cinnamate
FI: Fixed image
MI: Moving image
AEC: 3-Amino-9-ethyl carbazole
GCNIS: Germ cell neoplasia in situ

## References

[1] Darzi, F. & Bocklitz, T. A review of medical image registration for different modalities. *Bioengineering*10.3390/bioengineering11080786 (2024). 39199744 10.3390/bioengineering11080786 PMC11351674

[2] Báskay, J. et al. Reconstructing 3d histological structures using machine learning (artificial intelligence) algorithms. *Die Pathologie* **45**(1), 67–73. 10.1007/s00292-024-01387-6 (2024). 39570395 10.1007/s00292-024-01387-6

[3] Nolte, P. et al. Current approaches for image fusion of histological data with computed tomography and magnetic resonance imaging. *Radiology Research and Practice* **2022**(1), 6765895. 10.1155/2022/6765895 (2022). 36408297 10.1155/2022/6765895 PMC9668453

[4] Svetlove, A. et al. X-ray phase-contrast 3d virtual histology characterises complex tissue architecture in colorectal cancer. Frontiers in Gastroenterology **Volume 2 - 2023**10.3389/fgstr.2023.1283052 (2023). 10.3389/fgstr.2023.1283052 PMC12952374 41821788

[5] Albers, J. et al. Elastic transformation of histological slices allows precise co-registration with microCT data sets for a refined virtual histology approach. *Sci. Rep.* **11**(1), 10846 (2021). 34035350 10.1038/s41598-021-89841-w PMC8149420

[6] Chen, J. et al. 2d-3d deformable image registration of histology slide and micro-ct with disa-based initialization. Scientific Reports **15**(1), 10.1038/s41598-025-11583-w (2025). 10.1038/s41598-025-11583-w PMC12271352 40676123

[7] Nolte, P. et al. Spatial correlation of 2D hard-tissue histology with 3D microCT scans through 3D printed phantoms. *Scientific Reports* **13**(1), 18479. 10.1038/s41598-023-45518-0 (2023). 37898676 10.1038/s41598-023-45518-0 PMC10613209

[8] Museyko, O. et al. Registration of 2d histological sections with 3d micro-ct datasets from small animal vertebrae and tibiae. *Computer Methods in Biomechanics and Biomedical Engineering* **18**(15), 1658–1673. 10.1080/10255842.2014.941824 (2015). 25136982 10.1080/10255842.2014.941824

[9] Sarve, H., Lindblad, J., Borgefors, G. & Johansson, C. B. Extracting 3d information on bone remodeling in the proximity of titanium implants in srct image volumes. *Computer Methods and Programs in Biomedicine* **102**(1), 25–34. 10.1016/j.cmpb.2010.12.011 (2011). 21269725 10.1016/j.cmpb.2010.12.011

[10] Liu, J. T. C., Glaser, A. K., Poudel, C. & Vaughan, J. C. Nondestructive 3d pathology with light-sheet fluorescence microscopy for translational research and clinical assays. *Annual Review of Analytical Chemistry* **16**(1), 231–252. 10.1146/annurev-anchem-091222-092734 (2023). 36854208 10.1146/annurev-anchem-091222-092734 PMC12829911

[11] Pinkert-Leetsch, D. et al. First application of three-dimensional light sheet fluorescence microscopy to human testicular tumors: New perspectives in histopathology. *Andrology*10.1111/andr.70009 (2025). 39936385 10.1111/andr.70009 PMC12569739

[12] Reder, N. P. et al. Open-top light-sheet microscopy image atlas of prostate core needle biopsies. *Archives of Pathology & Laboratory Medicine* **143**(9), 1069–1075. 10.5858/arpa.2018-0466-oa (2019). 10.5858/arpa.2018-0466-OA PMC7402418 30892067

[13] Nolte, P. et al. Current approaches for image fusion of histological data with computed tomography and magnetic resonance imaging. *Radiology Research and Practice* **2022**, 1–20. 10.1155/2022/6765895 (2022). 10.1155/2022/6765895 PMC9668453 36408297

[14] Escobar Díaz Guerrero, R., Oliveira, J. L., Popp, J. & Bocklitz, T. Mmir: An open-source software for the registration of multimodal histological images. *BMC Medical Informatics and Decision Making* **24**(1), 65. 10.1186/s12911-024-02424-3 (2024). 38443881 10.1186/s12911-024-02424-3 PMC10916274

[15] Patterson, H. & Manz, T. NHPatterson/wsireg: Wsireg v0.3.5. *Zenodo*10.5281/ZENODO.6561996 (2022).

[16] Chiaruttini, N. et al. An open-source whole slide image registration workflow at cellular precision using fiji, qupath and elastix. *Frontiers in Computer Science*. **3**, 10.3389/fcomp.2021.780026 (2022).

[17] Unger, J., Sun, T., Chen, Y.-L., Phipps, J. E. & Bold, R. J. Method for accurate registration of tissue autofluorescence imaging data with corresponding histology: a means for enhanced tumor margin assessment. *Journal of Biomedical Optics* **23**(01), 1. 10.1117/1.jbo.23.1.015001 (2018). 29297208 10.1117/1.JBO.23.1.015001 PMC5749583

[18] Pinkert-Leetsch, D. et al. Three-dimensional analysis of human pancreatic cancer specimens by phase-contrast based x-ray tomography - the next dimension of diagnosis. *Cancer Imaging* **23**(1), 43. 10.1186/s40644-023-00559-6 (2023). 37131262 10.1186/s40644-023-00559-6 PMC10152799

[19] Frohn, J. et al. 3d virtual histology of human pancreatic tissue by multiscale phase-contrast x-ray tomography. *Journal of Synchrotron Radiation* **27**(6), 1707–1719. 10.1107/S1600577520011327 (2020). 33147198 10.1107/S1600577520011327 PMC7642968

[20] Sagar, M. M. R. et al. Optical clearing: an alternative sample preparation method for propagation based phase contrast ct. Frontiers in Physics **Volume 12 - 2024**, 10.3389/fphy.2024.1433895 (2024).

[21] GIMP — gimp.org. https://www.gimp.org/. [Accessed 05-05-2025].

[22] Bradski, G. The OpenCV Library. Dr. Dobb’s Journal of Software Tools (2000).

[23] McCormick, M., Liu, X., Jomier, J., Marion, C. & Ibanez, L. ITK: enabling reproducible research and open science. *Frontiers in Neuroinformatics* **8**, 13. 10.3389/fninf.2014.00013 (2014). 24600387 10.3389/fninf.2014.00013 PMC3929840

[24] Klein, S., Staring, M., Murphy, K., Viergever, M. A. & Pluim, J. P. W. elastix: a toolbox for intensity-based medical image registration. *IEEE Trans. Med. Imaging* **29**(1), 196–205 (2010). 19923044 10.1109/TMI.2009.2035616

[25] Shamonin, D. P. et al. Fast parallel image registration on cpu and gpu for diagnostic classification of alzheimer’s disease. *Frontiers in Neuroinformatics* **7**(50), 1–15 (2014). 10.3389/fninf.2013.00050 PMC3893567 24474917

[26] GitHub - ocornut/imgui: Dear ImGui: Bloat-free Graphical User interface for C++ with minimal dependencies — github.com. https://github.com/ocornut/imgui. Accessed 05-05-2025.

[27] GitLab - hawk-public/litshi:. https://gitlab.com/hawk-public/litshi. Accessed 16-12-2025.

[28] Flowchart Maker Online Diagram Software — app.diagrams.net. https://app.diagrams.net/. Accessed 05-05-2025.

[29] Hashemi, N. S., Aghdam, R. B., Ghiasi, A. S. B., & Fatemi, P. Template Matching Advances and Applications in Image Analysis. https://arxiv.org/abs/1610.07231 (2016).

[30] Sarvaiya, J. N., Patnaik, S. & Bombaywala, S. Image registration by template matching using normalized cross-correlation. In: *2009 International Conference on Advances in Computing, Control, and Telecommunication Technologies*, pp. 819–822. 10.1109/ACT.2009.207 (2009).

[31] Akl, S. G. & Toussaint, G. T. A fast convex hull algorithm. *Information Processing Letters* **7**(5), 219–222. 10.1016/0020-0190(78)90003-0 (1978).

[32] Fischler, M. A. & Bolles, R. C. Random sample consensus: a paradigm for model fitting with applications to image analysis and automated cartography. *Commun. ACM* **24**(6), 381–395. 10.1145/358669.358692 (1981).

[33] Mattes, D., Haynor, D. R., Vesselle, H., Lewellen, T. K. & Eubank, W. Pet-ct image registration in the chest using free-form deformations. *IEEE Transactions on Medical Imaging* **22**(1), 120–128. 10.1109/TMI.2003.809072 (2003). 12703765 10.1109/TMI.2003.809072

[34] Pluim, J. P. W., Maintz, J. B. A. & Viergever, M. A. Mutual-information-based registration of medical images: a survey. *IEEE Transactions on Medical Imaging* **22**(8), 986–1004. 10.1109/TMI.2003.815867 (2003). 12906253 10.1109/TMI.2003.815867

[35] Nandish, S., Prabhu, G. & Rajagopal, K. V. Multiresolution image registration for multimodal brain images and fusion for better neurosurgical planning. *Biomed. J.* **40**(6), 329–338. 10.1016/j.bj.2017.09.002 (2017). 29433836 10.1016/j.bj.2017.09.002 PMC6138619

[36] McCormick, M. M., Liu, X., Ibanez, L., Jomier, J. & Marion, C. Itk: enabling reproducible research and open science. *Front. Neuroinform.*10.3389/fninf.2014.00013 (2014). 24600387 10.3389/fninf.2014.00013 PMC3929840

[37] Lowe, D. G. Object recognition from local scale-invariant features. In: *Proceedings of the Seventh IEEE International Conference on Computer Vision*. vol. 2, pp. 1150–11572. 10.1109/ICCV.1999.790410 (1999).

[38] Schneider, C. A., Rasband, W. S. & Eliceiri, K. W. Nih image to imagej: 25 years of image analysis. *Nat. Methods* **9**(7), 671–675. 10.1038/nmeth.2089 (2012). 22930834 10.1038/nmeth.2089 PMC5554542

[39] Nolden, M. et al. The medical imaging interaction toolkit: challenges and advances. *International Journal of Computer Assisted Radiology and Surgery* **8**(4), 607–620. 10.1007/s11548-013-0840-8 (2013). 23588509 10.1007/s11548-013-0840-8

[40] Foret, P., Kleiner, A., Mobahi, H. & Neyshabur, B. Sharpness-Aware Minimization for Efficiently Improving Generalization. *arXiv* 2010.01412 (2020).

[41] Griebel, T., Archit, A. & Pape, C. Segment Anything for Histopathology. https://arxiv.org/abs/2502.00408 (2025).

