# Supplementary material for: Registration-based 3D Light Sheet Fluorescence Microscopy and 2D histology image fusion tool for pathological specimen
Source: Sci Rep. 2026 Jun 30;16:19915. doi: 10.1038/s41598-026-57893-5 (PMC13319223; doi:10.1038/s41598-026-57893-5)
Supplement: Supplementary file 1 — Supplementary Information. [file 41598_2026_57893_MOESM1_ESM.pdf]

Supplementary Figures

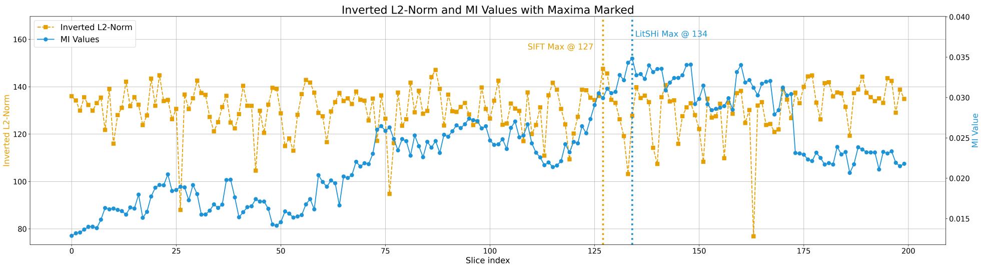

(A)

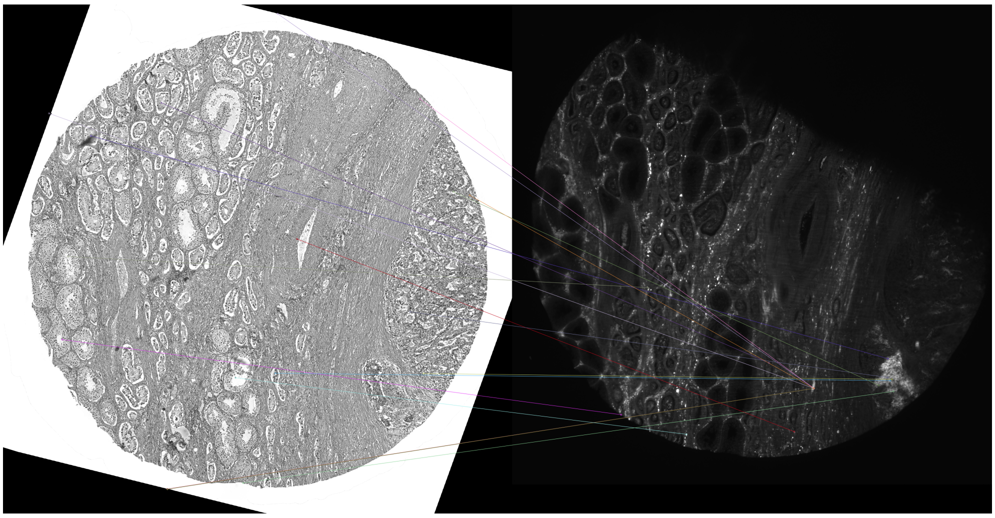

(B)

Fig. S1: (A) Comparison of the inverted L^2 norm computed from SIFT feature points in each layer of the LSFM stack and the histological slice, together with the mutual information values produced by our rotational template-matching approach. The L^2 norm was inverted to visualize the optimal plane as a maximum in the graph, analogous to the mutual information metric. (B) SIFT feature points detected in the stack plane (plane 127) with the highest inverted L^2 norm.

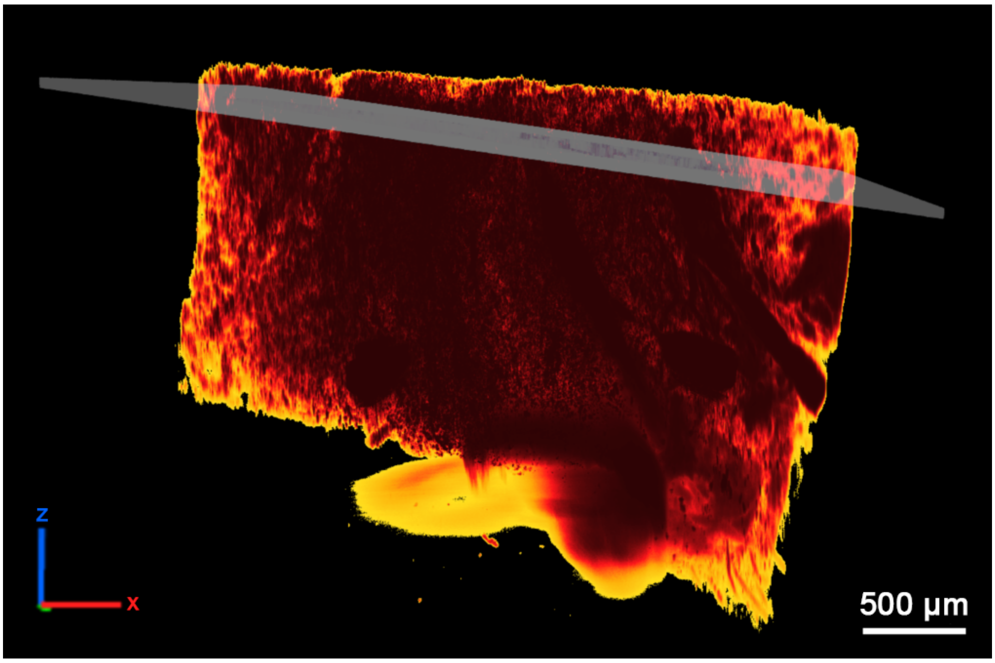

Fig. S2: 3D rendering of the histological plane used to identify corresponding patches (see Section 3.6) aligned within the LSFM stack. A red-to-yellow colormap was applied to the 3D scan to enhance the visibility of the plane, shown in white.
